# Detection, identification, and differentiation of sheep pox virus and goat pox virus from clinical cases in Giza Governorate, Egypt

**DOI:** 10.14202/vetworld.2016.1445-1449

**Published:** 2016-12-18

**Authors:** M. A. Mahmoud, M. H. Khafagi

**Affiliations:** Department of Parasitology and Animal Diseases, Veterinary Research Division, National Research Centre, Dokki 12622, Giza, Egypt

**Keywords:** Capripoxvirus, polymerase chain reaction, goat pox, isolation, RPO30 gene, sheep pox, transmission electron microscope

## Abstract

**Aim:**

To isolate, identify, and differentiate *Capripoxviruses* (CaPV) (sheep pox virus and goat pox virus) infections by egg inoculation, transmission electron microscopy (TEM), and 30 kDa RNA polymerase subunit gene-based polymerase chain reaction (PCR) (RPO30) in clinically affected animals in Hawamdia township of Giza Governorate, Egypt.

**Materials and Methods:**

A total of 37 scab samples were collected from clinically suspected field cases of sheep pox and goat pox. These samples were collected during (2014-2015) during different outbreaks of sheep pox and goat pox from Hawamdia township of Giza Governorate, Egypt. The samples were subjected to egg inoculation, TEM, and (RPO30) gene-based PCR. By using the egg inoculation: Previously prepared 37 scab samples (n=23 sheep and n=14 goats) were inoculated on the chorioallantoic membrane of specific pathogen free (SPF) embryonated chicken eggs (12 days old age). In the presence of the suitable percentage of humidity and candling, the inoculated eggs were incubated at 37°C. By using the TEM: Samples showed positive pock lesions on the chorioallantoic membranes, were fixed in glutaraldehyde, then processed and sectioned for TEM. Using the (RPO30) gene-based PCR assay, 30 of positive samples after egg inoculation (n=19 sheep and n=11 goats) were screened.

**Results:**

Using the egg inoculation, a characteristic pock lesions for poxviruses were seen in 30/37 (n=19 sheep and n=11 goats) (81.08%). Using the TEM, examination of the positive samples after egg inoculation revealed positive result in 23/30 (n=15 sheep and n=8 goats) (76.66%). The positive results represented by the presence of negatively stained oval-shape virus particles. Using the (RPO30) gene-based PCR assay, out of 30 total of positive samples after egg inoculation (n=19 sheep and n=11 goats) were screened, 27 (90%) samples (n=17 sheep and n=10 goats) were positive. The given band sizes of sheep and goats were 172 and 152 bp, respectively.

**Conclusion:**

PCR assay depended on RPO30 gene can be used lonely for the detection, identification, and differentiation of CaPVs. RPO30 gene-based PCR assay in combination with gene sequencing helps in molecular epidemiological studies of CaPV infection.

## Introduction

Sheeppox virus (SPPV) and goat pox virus (GTPV) belong to the Poxviridae family, genus *Capripoxvirus* (CaPV) which includes also lumpy skin disease virus (LSDV). Members of genus CaPV cause diseases in sheep, goats, and cattle, respectively. Members of genus CaPVs are closely related. The identity between the members of the same genus is 96% and 99% of isolates of the same species [1].

Clinically and serologically, we could not differentiate between the members of the genus (CaPVs) [2-4]. Sheep pox and goat pox not only tend to be host specific but also have the ability to infect naturally or experimentally both goats and sheep and cause diseases, respectively [5-7].

Members of genus Capripox cause transboundary diseases, so the World Organization for Animal Health categorizes it as notifiable diseases [3]. Sheep pox and goat pox have worldwide distribution and endemic throughout southwest and central Asia, northern and central Africa, and Middle East countries including Egypt [8,9].

Transmission of SPPV and GTPV occurs by direct and indirect contact to aerosols, respiratory droplets or contact with oronasal secretions produced by acutely infected animals [4,6]. Transmission through contact exposure with abrasions or mechanical transmission by arthropod vectors may also occur [10].

GTPV and SPPV infections give clear clinical signs, in the form of high body temperature, anorexia, depression, inflammation of the mucous membranes of the eyes and nose, respiratory distress, different stages of skin lesions (from erythema to scabs), and enlargement of superficial lymph nodes [10,11].

Throughout the entire course of infection, a transient viremia may not be clear. Papules could be seen at necropsy especially in the lungs, the upper respiratory tract, rumen, the upper digestive tract, kidney, and reproductive organs [2].

These viruses are responsible for the significant economic impact. Young animals are the most susceptible. The mortality rates ranged between 50% and 70% due to pneumonia [12,13]. Controlling of outbreaks is via ring vaccination, quarantine, and slaughter. High economic losses are due to mortalities, reduced productivity, and trade restrictions [9,14].

Maksyutov *et al*. [8] reported that the current classification of CaPVs based on the animal species from which the viruses are isolated, and this suggests that CaPVs are strictly host-specific [8]. Several reports indicated that both GTPV and SPPV were involved together in some CaPV Outbreaks. Therefore, this classification is inaccurate [3,11]. Due to the inability to distinguish clinically or serologically between the CaPV infections, some researcher recommended the need to more reliable tests for virus identification based on molecular methods [15].

Because of the close antigenicity between SPPV and GTPV, we cannot differentiate these pathogens using serological tests [12]. Some authors reported the use of highly sophisticated methods to differentiate SPPV from GTPV. These methods include restriction endonuclease analysis, whole genome sequencing and P32 gene-based polymerase chain reaction-restriction fragment length polymorphism (PCR-RFLP) [15]. Several suitable molecular assays containing species-specific signatures developed for the detection of CaPVs [16-21]. Most of these tests are restricted to detect only one viral species [21]. Other tests need the use of species-specific primers on a multiplex basis [19]. The third group of tests consumes time for post-PCR processing step [16]. In addition, most of these tests validated for detecting limited numbers of CaPV strains.

RPO30 gene-based PCR depends on the presence of RPO30 gene encoding the 30 kDa RNA polymerase subunit in CaPVs [22-24]. Lamien *et al*. [16] concluded that the presence of a 21-nucleotide deletion in the RPO30 gene of SPPV strains and absent in GTPV strains is the base of differentiation between them.

In this study, we used PCR for amplification of 30 kD subunit gene to differentiate the two pathogens. The used samples were from clinical cases of pox disease outbreaks in Hawamdia township of Giza Governorate, Egypt.

## Materials and Methods

### Ethical approval

The experiments were conducted in accordance with the guidelines laid down by the International Animal Ethics Committee and in accordance with local laws and regulations.

### Samples

About 37 scab samples of suspected field cases of sheep pox and goat pox (n=23 sheep and n=14 goats) were collected. The animal flocks were resident in Hawamdia township of Giza Governorate, Egypt. These samples were collected from different outbreaks during 2014 and 2015. The samples were kept in −70°C deep freezer until used.

### Virus isolation

According to the method described by Mahmoud *et al*. [25], Scabs, which were formed over the face, fatty tail and the rest of the body of the affected animals, were collected as a source of the virus and used for egg inoculation. The scabs were ground in 0.1 M sterile phosphate-buffered saline (PBS) containing antibiotics (penicillin 100 U/ml, streptomycin 100 µg/ml, and kanamycin 50 µg/ml). The suspension was frozen and thawed 3 times, and then centrifuged at 2000 g for 30 min. Then, 0.1 ml of the supernatant was inoculated in SPF embryonated chicken eggs (12 days old age). Sterile PBS was inoculated in SPF embryonated chicken eggs (12 days old age) and used as negative control. Inoculated eggs were incubated at 37°C in the presence of the suitable percentage of humidity with candling. After 5 days, the allantoic fluids and chorioallantoic membranes (CAMs) were collected carefully. CAMs were examined for the presence of the characteristic pock lesions of Poxviridae family.

### Electron microscopy

Samples showed positive pock lesions on the CAMs, were fixed in glutaraldehyde, then processed and sectioned for transmission electron microscopy (TEM) (Joel EM 100 CX II at 30 Kv) in EM unit, National Research Centre and examined as described by Mahmoud *et al*. [25].

### DNA extraction

DNA was extracted from the collected CAMs with pock lesions by using QIAamp DNA extraction kit (QIAGEN). Infected CAMs were thoroughly homogenized, separately, in liquid nitrogen and 20 mg of tissue powder was placed in a 2 ml microfuge tube. Then, lysis buffer and proteinase K were added and incubated at 56°C in a water bath until complete lysis of the tissues. DNA was then extracted according to the manufacturer’s instructions, eluted with 100 µl elution buffer and stored at −20°C.

### PCR

The PCR protocol described by Cohen *et al*. [26], which based on the RPO30 gene to differentiate GTPV from SPPV was used. The forward PCR primer was SpGp RNA Pol F 5’-TCTATGTCTTGATATGTGGTGGTAG-3’ and the reverse primer is SpGp RNA Pol 5’-AGTGATTAGGTGGTGTATTATTTTCC-3’ (synthesized by IDT, USA). The test was carried out in a 200 µl capacity PCR tube. 12.5 µl Maxima Hot Start Green PCR Master Mix (Fermentas, USA), 0.5 µl of each primer (10 pmol/µl), 1 µl of extracted DNA, and 10.5 µl of nuclease free water. Nuclease-free water was used as negative control. The amplification conditions were initial denaturation at 95°C for 4 min, followed by 40 cycles of denaturation at 95°C for 30 s, annealing at 55°C for 30 s and extension at 72°C for 30 s and final extension at 72°C for 5 min in a thermocycler (Eppendorf AG, Hamburg, Germany). The PCR products were electrophoresed on 3% agarose gel for 1 h at 100 V.

## Results

By egg inoculation, SPPV and GTPV viruses were successfully isolated. Characteristic pock lesions for Poxviridae family were observed on the CAMs. The negative control showed no change. Egg inoculation revealed characteristic pock lesions for pox viruses in 30/37 (19 sheep and 11 goats) (81.08%). While the rest of the sample (7/37 [18.92%]) showed no pathological changes on the CAM (Table-1). Positive pock lesion samples were prepared for TEM examination.

**Table-1:** Results of isolation of SPPV and GTPV by inoculation on embryonated chicken egg, electron microscope, and 30 kDa RNA polymerase subunit (RPO30) gene-based PCR.

Samples	Total samples	Egg inoculation		Electron microscope		PCR	
		
		+ve/%	−ve	+ve/%	+ve	+ve/%	−v ve
Sheep samples	23	19 (82.60)	4	15 (78.94)	4	17	2
Sheep pox virus	Could not be differentiated					17 (89.47)	2
GTPV	Could not be differentiated					0	0
Goats samples	14	11 (78.57)	3	8 (72.72)	3	10	1
SPPV	Could not be differentiated					0	0
GTPV	Could not be differentiated					10 (90.90)	1
Total	37	30 (81.08)	7	23 (76.66)	7	27 (90.00)	3

SPPV=Sheep pox virus, GTPV=Goat pox virus, PCR=Polymerase chain reaction

**Figure-1:**
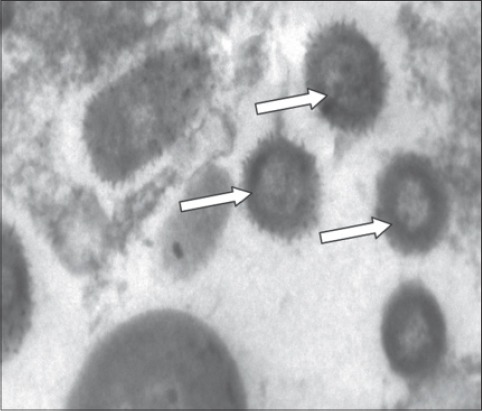
Arrows point to sheep poxviral particles visualized by transmission electron microscopy.

TEM examination of the positive samples after egg inoculation revealed positive result in 23/30 (76.66%) (15 sheep and 8 goats). The viral particles were oval shaped with negative staining. Protein filaments project from the external membrane. This arrangement is distinctive for members of CaPVs. The viral particles were approximately 130-260 nm in size at magnifications of 30,000 (Table-1 and Figure-1).

**Figure-2:**
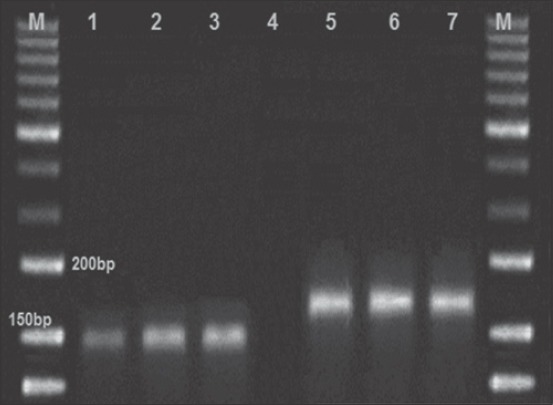
Results of polymerase chain reaction assay for differentiation of sheep pox virus (SPPV) and goat pox virus (GTPV). Lane (M): 50 bp ladder, Lane 1: SPPV positive control, Lane 2 and 3: SPPV positive samples, Lane 4: Control negative, Lane 5 and 6: GTPV positive samples, Lane 7: GTPV positive control.

About 30 positive CAM samples were subjected to viral DNA extraction. All these samples expressed pock lesion were screened with RPO30 gene based PCR and 27/30 (90%) of the tested samples were positive (17 sheep and 10 goats). Positive control for both sheep and goat pox was included. The other 3/30 positive CAM that gave the negative results with the PCR may be due to any mistake during the DNA extraction process. The appropriate fragment size for SPPV is 151 bp and for GTPV is 172 bp of the RPO30 gene-based PCR were obtained. The results revealed no cross infection in any of the tested animal samples (Table-1 and Figure-2).

## Discussion

The current classification of CaPVs based on the animal species from which the viruses are isolated, and this suggests that CaPVs are strictly host-specific [8]. This conviction was corroborated by the absence of CaPV-like disease in sheep or goats in endemic areas with LSD in cattle. Although some countries are endemic with sheep pox and goat pox, LSD did not report in it [27]. Moreover, diagnosis of pox in sheep and goats is often described clinically as only sheep pox or goat pox, respectively. Both sheep pox and goat pox are easily diagnosed clinically, but the causal agent cannot be differentiated at the field level [26].

Although many reports of capripox outbreaks occurred in sheep and goats and the causative agents were SPPVs and GTPVs [11]. In very rare cases, isolation of O-240 isolate of LSD from Kenyan sheep was reported [2]. For this reason, it is recommended to completely identify the causal virus in capripox outbreaks.

Various species of game animals could play a role in maintaining CaPV-like disease outbreaks but still suggestion [15]. Phylogenetic analysis of E4L gene in the CaPV led to three different clusters, LSDV, GTPV and SPPV [1,15,16,26]. Lamien *et al*. [16] reported that the incidence of LSDV in small ruminants appears to be extremely rare, so the use of CaPVs RPO30 PCR allows the fast differentiation of SPPV and GTPV infecting small ruminants without the need of gene sequencing [26]. Unlike GTPV and LSDV, SPPV group members only have a 21-nucleotide deletion in the RPO30 gene [26].

In this study, we could detect and differentiate 27 positive samples (sheep n=17 and goats n=10) for SPPVs and GTPVs, respectively, in actually short time with low cost without the need of using of species-specific primers on a multiplex basis [19] or consuming the time for post-PCR processing steps [17]. In addition, this test allowed screening of large numbers of samples in very short time.

The time consumed in samples preparation, egg inoculations, and TEM is about 12 days. After applying egg inoculation and TEM, the samples were still undifferentiated. This time is enough for detection and differentiation of multiple numbers of the samples if we use CaPVs RPO30 PCR method. In addition, the CaPVs RPO30 PCR could be applied on the scab samples directly without the step of egg inoculation [28].

By using the TEM on 30 samples (sheep n=19 and goats n=11) which gave positive results by the egg inoculations, there were only 23 positive results (sheep n=15 and goats n=8) as shown in Table-1 and Figure-1. After testing the above 30 samples with CaPVs RPO30 PCR method, 27 positive results (sheep n=17 and goats n=10) were obtained. TEM succeeded in the detection of 23/30 (76.66%) of the samples without differentiation of SPPV and GTPV. CaPVs RPO30 PCR succeeded in the detection of 27/30 (90.0%) of the samples with differentiation of SPPV and GTPV as shown in Table-1 and Figure-2. Therefore, it is advisable to apply the CaPVs RPO30 PCR rather than the traditional methods used above to avoid the inaccurate diagnosis of capripox outbreaks.

The variations between the results of the two diagnostic techniques used represented by sheep 2/19 (10.0%) and goats 2/11 (18.18%). It may be due to an inaccurate selection of the visualized slides or improper preparation of the samples. In addition, the CaPVs RPO30 PCR method is more sensitive technique than TEM technique.

RPO30 gene present in both SPPV and GTPV but the lengths of both varies due to the existence of a 21-nucleotide deletion in the 5’ end in SPPV and absent in GTPV. In our study, we used a primer set that amplifies partially and completely the RPO30 gene in SPPV and GTPV, respectively. The amplicon size of SPPV is 152 bp while the amplicon size of GTPV (172 bp) and this is compromised with the results obtained by Cohen *et al*. [26] and Yan *et al*. [29]. In the past, detection and identification of CaPVs were mentioned in several PCR assays [4,14-17]. Most of these assays did not go beyond the genus level.

In this study, all the positive samples of sheep pox and goat pox were from sheep and goats, respectively. This means there is no cross infection. Unlike P32 gene-based PCR-RFLP [4,16], multiplex PCR [10]. RPO30 gene-based PCR is simple, accurate and cheap for differentiation of SPPV and GTPV in a single PCR.

In our study, we used only one set of primers, and we succeeded in the detection of the characteristic fragments of both SPPVs and GTPVs. The CaPVs RPO30 PCR method did not need the use of any genotype-specific primers. In addition to the ability of the CaPVs RPO30 PCR to be used in screening and genotyping of CaPVs during outbreaks in sheep and goats if sequencing is accompanied [26].

## Conclusion

PCR assay depended on RPO30 gene can be used lonely for detection and identification of CaPVs infection. In this study, all of the tested samples gave amplicon’s size that is in agreement with their host origin. The cross infection did not take apart in this study. This may be due to the low number of the used samples, but in the case of huge numbers of samples, the cross-infection may appear. With the use of sequencing, RPO30 gene-based PCR assay can give a good picture of molecular epidemiology of CaPV infection around the world.

## Authors’ Contributions

MAM and MHK conceived the study, performed the fieldwork, collected the samples, carried out the laboratory work, analyzed the data, drafted the manuscript, and read and approved the final manuscript.
